# Sustained clinical remission under infliximab/rituximab combination therapy in a patient with granulomatosis with polyangiitis

**DOI:** 10.1186/s13317-020-00147-9

**Published:** 2021-03-06

**Authors:** Larissa Valor-Méndez, Arnd Kleyer, Jürgen Rech, Bernhard Manger, Georg Schett

**Affiliations:** 1 Department of Internal Medicine 3, Rheumatology and Immunology, Friedrich Alexander University Erlangen-Nuremberg and Universitätsklinikum Erlangen, Ulmenweg 18, 91054 Erlangen, Germany; 2 Deutsches Zentrum für Immuntherapie (DZI) FAU Erlangen-Nürnberg and Universitätsklinikum Erlangen, Ulmenweg 18, 91054 Erlangen, Germany

**Keywords:** Granulomatosis with polyangiitis (GPA), Infliximab, Rituximab, Combined therapy

## Abstract

**Background:**

Granulomatosis with polyangiitis (GPA) is a systemic autoimmune disease characterized by small and medium vessel vasculitis. The use of biological therapies such as rituximab and infliximab has improved the treatment of ocular manifestations in GPA.

**Case report:**

We report a case of a 45-year-old Caucasian male suffering with rhinitis, sinubronchitis and exophthalmos. These clinical findings, subsequent biopsy and MRI were consistent with positive anti-neutrophil cytoplasm antibody (ANCA)/proteinase-3 and he was diagnosed with GPA with orbital involvement. He was refractory to cyclophosphamide at stable doses of methotrexate and a therapy with rituximab was started. Eventually and because of family planning methotrexate was replaced by azathioprine. Symptoms worsened and MRI revealed an increase in the granulomatous lesion in the orbit. Therefore, we decided to add infliximab to the combination of azathioprine and rituximab, our patient achieved then a long-term response. During the 10 years of the combined treatment, no adverse effects or systemic involvement occurred.

**Conclusions:**

This case suggests that the individual use of a combination of rituximab and infliximab may be a promising strategy for the treatment in the long term of refractory orbital GPA.

## Background

Granulomatosis with polyangiitis (GPA) is a systemic autoimmune disease with unidentified etiology characterized by small/medium vessel vasculitis, granulomatous inflammation and tissue necrosis. Its clinical presentation is highly variable and severe cases with organ involvement are not uncommon. Among them, eye complications occur in 50–60 % of patients and usually present as necrotizing scleritis or granulomatous orbital involvement. Orbital GPA can cause significant morbidity and potentially lead to complete loss of vision and long-lasting facial deformity [1, 2].

## Case presentation

We report a 45-year-old Caucasian male diagnosed with GPA in 2001 presenting with rhinitis, sinubronchitis and a right exophthalmos, but not signs of diplopia or vision defects. The nasal mucosa showed in two different biopsies active inflammation characterized by the presence of neutrophils and active vasculitis, tissue necrosis, and granulomatous inflammation. In the lacrimal ducts, a partially scarred tissue with moderate to severe chronic signs of inflammation was observed. Lab testing revealed anti-neutrophil cytoplasmic antibodies (cANCA) and against proproteinase-3 (anti-PR3). The patient was a non-smoker and had an unapparent medical history. No signs of kidney, lung, and joint, skin or central nervous system involvement were found.

The patient met the 1990 American College of Rheumatology (ACR) classification criteria for GPA [3]. Based on the clinical practice in 2001, when rituximab was not yet available for the treatment of GPA, intermittent cycles of cyclophosphamide at 15 mg/kg every month were started and the patient later received stable doses of methotrexate (15 mg/week) and low doses of glucocorticoids (5 mg daily) as maintenance treatment. In 2010 symptoms worsened, with increasing complaints related to inflammation of the upper airways (rhinitis, sinusitis and bronchitis) requiring hospital admission and the start of high-dose steroids (1 g/day pulsed methylprednisolone for 3 days, then 1 mg/kg/day for further 10 days. Cyclophosphamide (cumulative doses of 7 g) was stopped Rituximab was initiated at doses of 1000 mg at weeks 0–2 and then 500 mg every 6 months thereafter.

GPAthen remained silent with rituximab, methotrexate (15 mg/week) and low-dose prednisolone (5 mg daily) over 1-year. Because of family planning, methotrexate was replaced by azathioprine (150 mg/day), prednisolone was stopped and rituximab was continued. Three months later, the patient presented with persistent frontal headache, protrusion of the right eyeball and increased eye watering. Conventional MRI showed a granulomatous mass in the area of the right orbita, with granulomatous changes in the left lacrimal gland, indicating progression of disease despite rituximab treatment and almost complete B-lymphocyte depletion.

**Fig. 1:**
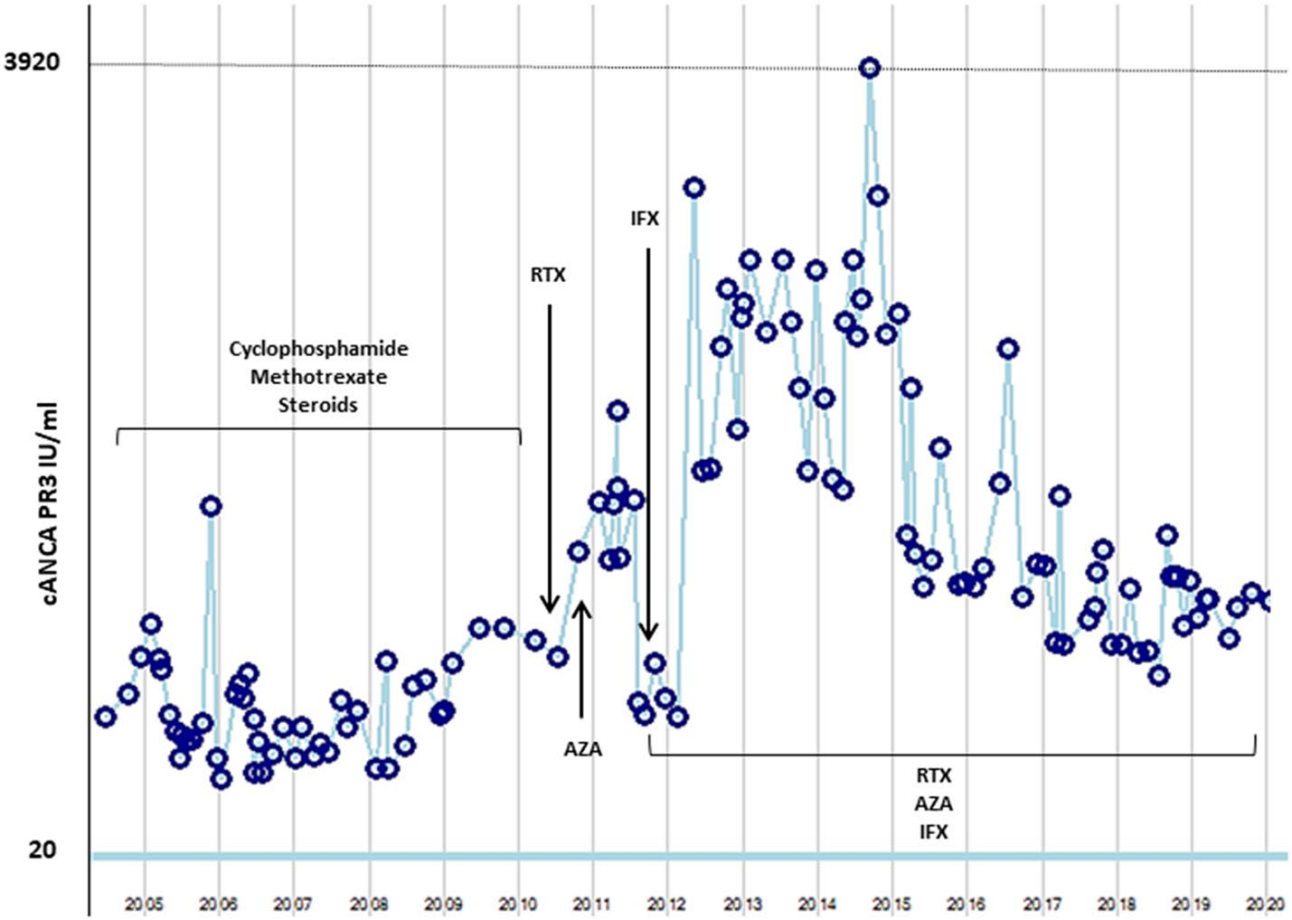
cANCA/PR3 levels in the last 15 years of treatment. The dotted line indicates the upper limit of the reached cANCA/PR3, the blue line indicates the upper limit of the normal range for cANCA/PR3. *RTX* rituximab, *IFX* infliximab, *AZA* azathioprin

In agreement with the patient and considering the substantial granuloma formation in the orbita infliximab was added to rituximab at 400 mg IV at weeks 0–2–6 and every 6 weeks thereafter. A remarkable improvement in symptoms and inflammation imaging after a 6-month follow-up was observed. This combination regimen of azathioprine, rituximab and infliximab was maintained and the patient achieved long-standing remission without infectious complications. A trimethoprim/sulfamethoxazole prophylaxis has been maintained for the last 10 years, which potentially mitigated infections. No adverse events occurred during the entire 10 years of treatment. Despite remission, ANCA remained consistently elevated during this time (Fig. 1), even considering the methods change for ANCA tests, from ELISA to chemiluninescence technique, which has been standardized and validated in our center according to international guidelines.. Currently, the patient has no complaints, the treatment has been well tolerated and MRI revealed only minimal residual lesions (3 mm in size) in the orbita in the 2019 follow-up.

## Conclusions

Rituximab has strongly improved the treatment of GPA including eye manifestations [4–6]. However, in our particular case rituximab treatment had not suffice. Our patient initially responded to rituximab but then showed recurrence of disease despite almost complete B lymphocyte depletion. Based on the granulomatous lesion in the orbita leading to exophthalmus we decided to add infliximab to rituximab and achieved long-term response. In two previous publications it was reported, that a patient diagnosed with GPA received a sequential treatment with cyclophosphamide, then with infliximab and, due to recurrence of symptoms with rituximab [7]. In another patient with a delayed diagnosis of GPA due to an unusual presentation, sequential treatment with steroids and infliximab was started, and then due to relapse the patient was treated with three courses of cyclophosphamide and a posteriori with rituximab [8].

This case report suggests that the use of a combination of rituximab and infliximab might be a promising strategy for the treatment of refractory GPA, the combination treatment has been well tolerated for a long time under tight monitoring and did not trigger an immune state that precipitates adverse events. We are aware of the fact that the use of two biological agents might imply an increased risk life-threatening infections or malignancies. It has been reported in 2012 that increased risk of both solid and hematologic malignancies in ANCA associated vasculitis, this publication states that the risk might be both, inherent to the disease or to the treatment, which is difficult to determine [9].

## Data Availability

Not applicable.
